# Avançando no Uso de Anticoagulantes Orais Diretos no Manejo do Trombo Ventricular Esquerdo

**DOI:** 10.36660/abc.20240409

**Published:** 2024-08-07

**Authors:** Pedro E. P. Carvalho

**Affiliations:** 1 Minneapolis Heart Institute Foundation Minneapolis EUA Center for Coronary Artery Disease - Minneapolis Heart Institute Foundation, Minneapolis - EUA

**Keywords:** Inibidores do Fator Xa, Tromboembolia Venosa, Infarto do Miocárdio

Os anticoagulantes orais diretos (DOACs) são preferidos aos antagonistas da vitamina K (AVKs) em vários cenários clínicos, como na prevenção de eventos tromboembólicos em pacientes com fibrilação atrial e no tratamento de tromboembolismo venoso.^1–4^ Em contraste, os DOACs são contraindicados em pacientes com válvulas cardíacas mecânicas, FA valvar devido a estenose mitral e síndrome antifosfolípide.^5–7^ Em pacientes com trombo ventricular esquerdo (TVE), entretanto, a eficácia e segurança dos DOACs permanecem incertas.

TVE é uma complicação comum após um infarto agudo do miocárdio (IAM). A incidência de TVE pós-IAM agudo diminuiu devido ao aumento da intervenção coronária percutânea primária (ICP) e terapias antitrombóticas avançadas. Na era da ICP primária, a incidência de TVE é de até 6,3% em pacientes com IAMCSST, aumentando para 19,2% naqueles com IAMCSST anterior e fração de ejeção ventricular esquerda reduzida.^8^ Dados do registro SWEDEHEART indicaram uma incidência de até 38%, sugerindo subestimação e necessidade de acompanhamento para avaliar a formação de TVE nesses pacientes.^9^ Além disso, a escolha da modalidade de imagem impacta significativamente a detecção de TVE, com a ressonância magnética cardíaca tendo maior sensibilidade em comparação com a ecocardiografia.^10^ Portanto, existe uma necessidade não atendida de prevenção, detecção e tratamento de TVE.

Quando presente, o TVE está associado a um alto risco de acidente vascular cerebral e embolia sistêmica, mesmo após tratamento anticoagulante. O manejo é desafiador devido à falta de dados randomizados com poder suficiente para abordar esta questão clínica. Atualmente, não há recomendação de rotina para profilaxia de TVE pós-IAM agudo. Um pequeno ECR demonstrou que doses baixas de rivaroxabana combinada com terapia antiplaquetária dupla (TAPD) versus TAPD isolada reduziram a incidência de TVE em 30 dias sem aumentar o sangramento maior.^11^ O ensaio APERITIF em andamento (NCT05077683) pretende randomizar mais de 500 participantes para analisar também se a adição de DOAC à TAPD é segura e eficaz na prevenção de TVE.

Os dados tradicionalmente apoiam o uso de AVKs para tratar TVE estabelecida, apesar do aumento do uso off-label de DOACs. Recentemente, uma declaração científica da AHA considerou os DOACs uma alternativa razoável aos AVK.^12^ Nesta edição dos Arquivos Brasileiros de Cardiologia é apresentada uma metanálise comparando AVKs com DOACs em pacientes com TVE.^13–15^ Esta análise incluiu quatro pequenos ensaios clínicos randomizados (ECR) e 29 estudos observacionais, abrangendo 4.450 pacientes. Os resultados indicam taxas de eventos tromboembólicos semelhantes entre AVKs e DOACs, com a rivaroxabana mostrando uma redução nos eventos tromboembólicos na análise de subgrupo em comparação com AVKs.

Esta metanálise oportuna apoia o uso de DOACs em pacientes com TVE, incluindo um estudo randomizado adicional e vários estudos observacionais não considerados na declaração da AHA. Os autores concluíram que não houve diferença significativa nos resultados hemorrágicos entre DOACs e AVKs, mas foi observado um efeito protetor dos DOACs em vários resultados hemorrágicos, sugerindo que a falta de significância estatística não deveria implicar uma falta de diferença clínica.^13^ Especialmente considerando que os AVKs estão associados a interações medicamentosas e alimentares, dose-resposta imprevisível e uma faixa terapêutica estreita, necessitando de monitoramento rigoroso do tempo de protrombina.^14^

Dada a natureza observacional da maioria dos estudos incluídos, existem preocupações sobre viés de seleção e variáveis de confusão, limitando a interpretação dos resultados. Devido ao desafio de alocar grandes coortes de pacientes com TVE em ECRs, é improvável obter dados randomizados em um futuro próximo. Este estudo, no entanto, orienta a extensão do uso de DOAC nesta população de pacientes, enfatizando a necessidade de evidências adicionais para melhorar os resultados clínicos em pacientes com TVE.^15^
