# Supplementary material for: Avançando no Uso de Anticoagulantes Orais Diretos no Manejo do Trombo Ventricular Esquerdo
Source: Arq Bras Cardiol. 2024 Aug 7;121(7):e20240409. [Article in Portuguese] doi: 10.36660/abc.20240409 (PMC11495807; doi:10.36660/abc.20240409)
Supplement: Translation

# Translation: Advancing the Use of Direct Oral Anticoagulants in Left Ventricular Thrombus Management

**DOI:** 10.36660/abc.20240409i

**Authors:** Pedro E. P. Carvalho

**Affiliations:** 1 Minneapolis USA Center for Coronary Artery Disease - Minneapolis Heart Institute Foundation, Minneapolis – USA

**Keywords:** Factor Xa Inhibitors, Venous Thromboembolism, Myocardial Infarction

Direct oral anticoagulants (DOACs) are preferred over Vitamin K Antagonists (VKAs) in numerous clinical scenarios, such as preventing thromboembolic events in patients with atrial fibrillation and treating venous thromboembolism.^1–4^ In contrast, DOACs are contraindicated in patients with mechanical heart valves, valvular AF due to mitral stenosis, and antiphospholipid syndrome.^5–7^ In patients with left ventricular thrombus (LVT), however, the efficacy and safety of DOACs remain uncertain.

LVT is a common complication following an acute myocardial infarction (MI). The incidence of LVT post-acute MI has declined due to increased primary percutaneous coronary intervention (PCI) and advanced antithrombotic therapies. In the primary PCI era, LVT incidence is up to 6.3% in STEMI patients, rising to 19.2% in those with anterior STEMI and reduced left ventricular ejection fraction.^8^ Data from the SWEDEHEART registry indicated an incidence as high as 38%, suggesting underestimation and the need for follow-up to assess LVT formation in these patients.^9^ In addition, the choice of imaging modality significantly impacts LVT detection, with cardiac magnetic resonance having higher sensitivity compared with echocardiography.^10^ Therefore, there is an unmet need for LVT prevention, detection, and treatment.

When present, LVT is associated with a high risk of stroke and systemic embolism, even post anticoagulant treatment. Management is challenging due to a lack of sufficiently powered randomized data addressing this clinical issue. Currently, there is no routine recommendation for LVT prophylaxis post-acute MI. A small RCT demonstrated that low-dose rivaroxaban combined with dual antiplatelet therapy (DAPT) versus DAPT alone reduced LVT incidence within 30 days without increasing major bleeding.^11^ The ongoing APERITIF trial (NCT05077683) aims to enroll over 500 participants to also analyze whether the addition of DOAC to DAPT is safe and effective in preventing LVT.

Data traditionally support the use of VKAs to treat established LVT despite the increasing off-label use of DOACs. Recently, an AHA scientific statement deemed DOACs a reasonable VKA alternative.^12^ In this issue of the *Arquivos Brasileiros de Cardiologia*, a meta-analysis comparing VKAs with DOACs in LVT patients is presented.^13–15^ This analysis included four small randomized controlled trials (RCTs) and 29 observational studies, encompassing 4,450 patients. The findings indicate similar thromboembolic event rates between VKAs and DOACs, with rivaroxaban showing a reduction in thromboembolic events compared with VKAs in a subgroup analysis.

This timely meta-analysis supports the use of DOACs in patients with LVT by including an additional randomized study and numerous observational studies not considered in the AHA statement. The authors concluded there was no significant difference in bleeding outcomes between DOACs and VKAs, but a protective effect of DOACs in several bleeding outcomes was observed, suggesting that the lack of statistical significance should not imply a lack of clinical difference.^13^ Especially considering that VKAs are associated with drug and food interactions, unpredictable dose-response, and a narrow therapeutic range, necessitating close monitoring of prothrombin time.^14^

Given the observational nature of most included studies, there are concerns about selection bias and confounding, limiting results interpretation. Due to the challenge of enrolling large LVT patient cohorts in RCTs, obtaining randomized data is unlikely soon. This study, however, guides extending DOAC use in this patient population, emphasizing the need for additional evidence to improve clinical outcomes in patients with LVT.^15^
